# Panhypopituitarism as the first sign of paraneoplastic limbic encephalitis in a patient with cured testicular cancer: a case report

**DOI:** 10.1186/s13256-022-03603-4

**Published:** 2022-09-29

**Authors:** Yvonne Lei, Alexandra Milin Glaeser

**Affiliations:** 1 University of California Los Angeles David Geffen School of Medicine, 10833 Le Conte Ave, Los Angeles, CA 90095 USA; 2 Department of Medicine, University of California Los Angeles David Geffen School of Medicine, Los Angeles, CA USA

**Keywords:** Paraneoplastic syndrome, Testicular cancer, Panhypopituitarism, Limbic encephalitis, Case report

## Abstract

**Background:**

Paraneoplastic limbic encephalitis is a rare neurologic syndrome that affects patients with cancer and commonly presents with symptoms of personality changes, visual disturbances, seizures, vertigo, and memory loss. It has an immune-mediated pathophysiology and is associated with antibodies directed against both the tumor and limbic structures in the nervous system. Here, we report a case of paraneoplastic limbic encephalitis with an unusual presentation with initial symptoms of panhypopituitarism.

**Case presentation:**

A 28-year-old Caucasian man with history of testicular cancer in remission for almost 2 years was admitted to our hospital for altered mental status, syncope, vertical gaze palsy, ataxia, and tremor. Three months prior to admission, he began to have initial symptoms of fatigue, weight gain, and hypogonadism. A few weeks before admission, he also developed worsening neurological symptoms that led him to present to the hospital. While hospitalized, he had an episode of syncope. Evaluation of his syncope revealed that he was hypovolemic due to polyuria, concerning for diabetes insipidus. While the patient did not meet criteria for diabetes insipidus, further endocrine laboratory testing to evaluate his central hormonal axes revealed panhypopituitarism. He also underwent a neurologic work-up with brain magnetic resonance imaging and lumbar puncture, with results consistent with encephalitis. He received high-dose steroids, plasmapheresis, and intravenous immunoglobulin, which did not significantly improve his physical examination or mental status. Extensive testing did not show any recurrence of his testicular cancer. Paraneoplastic antibodies were also ordered, with anti-Ma2 antibodies eventually returning positive, consistent with anti-Ma2 paraneoplastic limbic encephalitis.

**Conclusion:**

Paraneoplastic limbic encephalitis is often a difficult diagnosis due to its variable presentation and timeline of presentation, leading to delays in both diagnosis and treatment. While paraneoplastic limbic encephalitis usually presents with neurological symptoms, it may also present with panhypopituitarism. A high index of suspicion is warranted for paraneoplastic syndromes in patients with history of malignancy, even if the malignancy is in remission.

## Background

Paraneoplastic neurologic syndromes (PNS) are rare disorders that affect around 0.01% of patients with cancer and can act on any part of the nervous system [1]. For example, some types affect a single area of the brain (e.g., limbic encephalitis) or a single cell type (e.g., Purkinje cells). These paraneoplastic disorders are associated with antibodies directed against antigens expressed by both the tumor and the nervous system, indicating an immune-mediated pathophysiology. Of note, these disorders usually occur before the malignancy has been identified [1].

Paraneoplastic limbic encephalitis (PLE) is a subtype of PNS that can present with personality changes, visual disturbances, seizures, vertigo, and memory loss. It selectively affects limbic structures, including the hippocampus, hypothalamus, and amygdala. PLE has been most associated with small cell lung cancer, testicular cancer, and breast cancer [2]. In testicular cancer, PLE is most often associated with anti-Ma2 antibodies [1].

## Case presentation

A 28-year-old Caucasian man with history of testicular cancer in remission presented with altered mental status, syncope, vertical gaze palsy, ataxia, and tremor. He was in his usual state of health until 2 years ago, when he noticed a large right testicular mass. Work-up revealed marginally enlarged intraabdominal lymph nodes without evidence of metastases. One and a half years ago, he underwent radical unilateral orchiectomy. Pathology revealed malignant mixed germ cell tumor with abundant necrosis, comprising 50% teratoma, 45% embryonal carcinoma, and 5% yolk sac tumor. The mixed germ cell tumor was limited to the testicle without evidence of invasion. Lymph nodes were not examined pathologically. Alpha fetal protein (AFP) was mildly elevated at 9 ng/mL (reference range: 0–6.7 ng/mL) and normalized postoperatively. The patient opted for surveillance instead of chemotherapy after consultation with an oncologist.

Three months prior to admission, the patient started experiencing fatigue and weight gain of 18 kg over 2 months. He was diagnosed with hypogonadism based on a low testosterone level of 127 ng/dL and started testosterone supplementation. Three weeks prior to admission, he began experiencing difficulty with upward and downward gaze. His fatigue continued to worsen, and he stopped working. One week prior to admission, he developed confusion, which manifested in difficulty remembering his dogs’ names and asking questions in strangely worded ways. He also developed bilateral tremor and ataxia, causing him to fall on a daily basis.

The worsening of these neurological symptoms led him to present to the emergency department. While hospitalized, he had an episode of syncope. As this was being evaluated, he was noted to have hypovolemia due to polyuria, which was concerning for diabetes insipidus and, consequently, hypothalamic involvement of his underlying disease. Ultimately, the patient did not meet criteria for diabetes insipidus but did have elevated prolactin of 52.0 ng/mL, concerning for some hypothalamic injury. Due to suspicion that his condition could extend to the pituitary, the patient underwent testing for thyroid-stimulating hormone (TSH), luteinizing hormone (LH), follicle-stimulating hormone (FSH), and adrenocorticotropic hormone (ACTH). The decreased levels of the above hormones revealed probable panhypopituitarism, with TSH of 0.11 mcIU/mL, free T4 of 1.0 ng/dL, ACTH of <2 pg/mL, LH of 0.5 pg/mL, and FSH of 1.3 mIU/mL. Of note, he was placed on high-dose steroids immediately and has consistently been on these higher-dose steroids, so he has been unable to undergo a cosyntropin test to confirm central adrenal insufficiency.

**Fig. 1:**
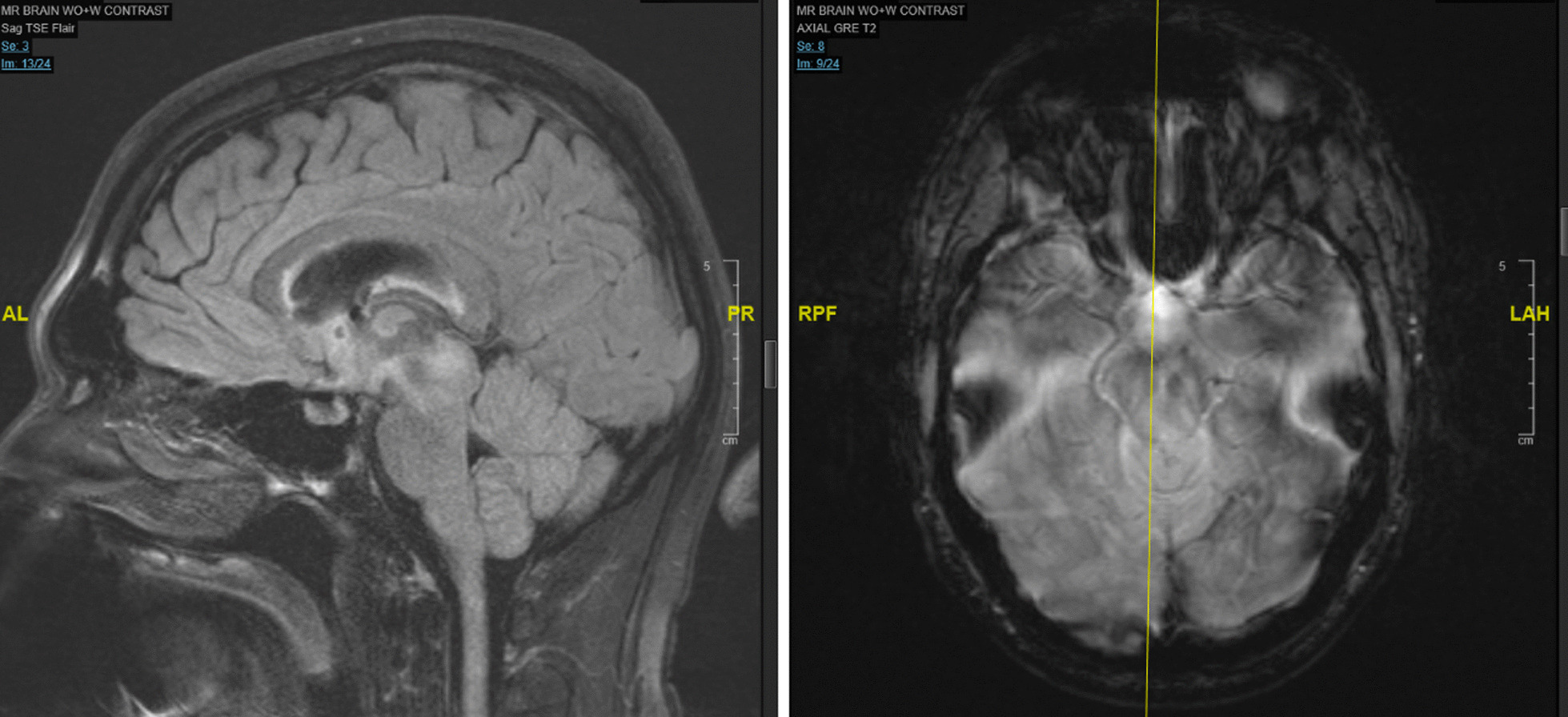
Initial brain magnetic resonance imaging showing T2 prolongation involving the bilateral mesial temporal lobes, hippocampi/fornices, hypothalamus, mammillary bodies and mammillothalamic tracts, and midbrain including periaqueductal gray

**Fig. 2:**
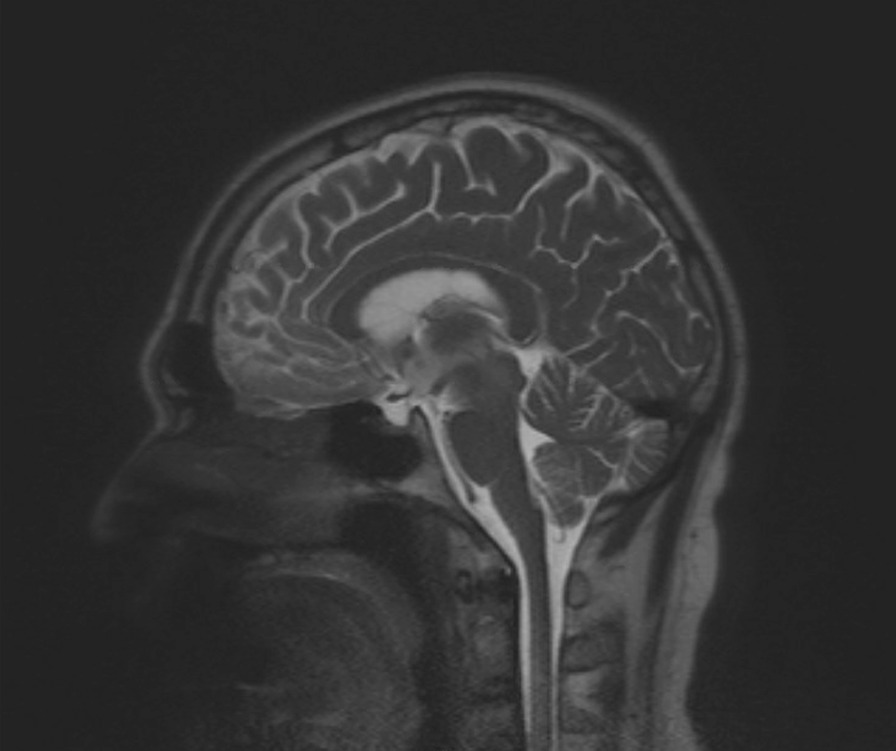
Magnetic resonance imaging of pituitary and brain showing symmetric T2/fluid-attenuated inversion recovery (FLAIR) hyperintensity in the mesial temporal lobes, hippocampi, hypothalamus, mamillary bodies, and midbrain including periaqueductal gray matter. Sella turcica and pituitary gland are normal in size

He also underwent a neurologic work-up, including brain magnetic resonance imaging (MRI) and MRI imaging of the pituitary, which showed T2 prolongation involving the bilateral mesial temporal lobes, hippocampi/fornices, hypothalamus, mammillary bodies and mammillothalamic tracts, and midbrain including periaqueductal gray with normal pituitary (Figs. 1 and 2). His presentation was concerning for encephalitis involving subcortical white matter, deep brain structures, and dorsal midbrain. A lumbar puncture showed lymphocytic pleocytosis with elevated protein and was positive for oligoclonal bands. A broad infectious work-up was otherwise negative. He received high-dose steroids, five sessions of plasmapheresis, and 5 days of intravenous immunoglobulin, without significant improvement in his physical examination or mental status. Repeat ultrasound of the testes, positron emission tomography (PET)/computed tomography (CT) scans, and AFP levels were all normal without evidence of recurrent testicular cancer. He was discharged with close follow-up, and eventually anti-Ma2 antibodies returned positive, consistent with anti-Ma2 PLE.

**Fig. 3:**
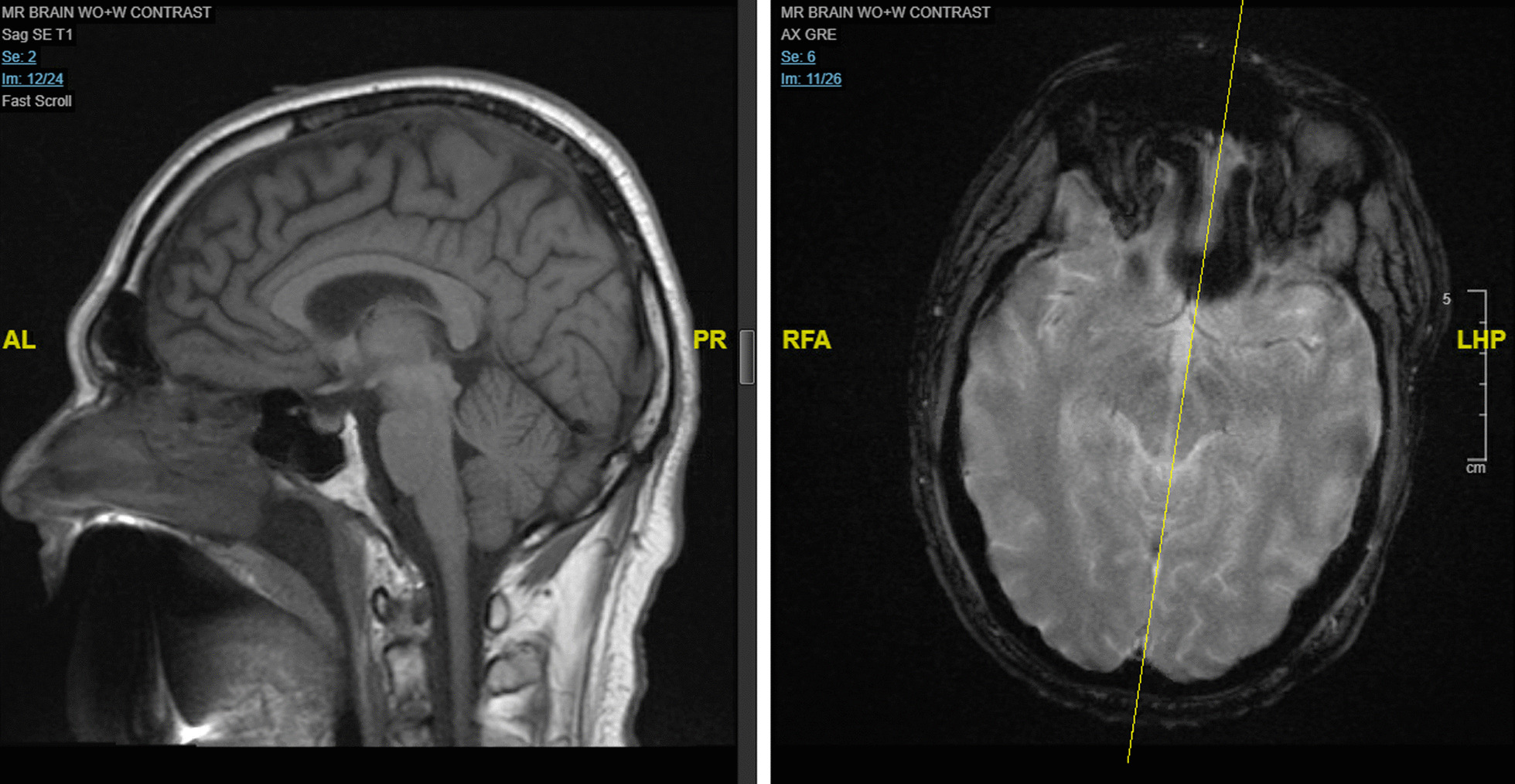
Brain magnetic resonance imaging 5 months after initial hospitalization, showing areas of signal abnormality involving the mesial temporal lobes, maxillary bodies, midbrain, and periaqueductal gray matter, which are overall similar in extent to prior brain MRI but mildly decreased in intensity

After discharge, the patient was treated with rituximab. Unfortunately, the patient did not make significant improvement after his hospitalization, losing the ability to walk and to clear his nasopharyngeal secretions. A few months later, the patient was readmitted to the hospital and intensive care unit (ICU) for progression of his anti-Ma2 PLE, which was complicated by thrombocytopenia, gastrointestinal (GI) bleed, sepsis, and worsening neurological status. During this hospitalization, the patient underwent tracheostomy and placement of a gastric tube. Repeat brain MRI showed similar areas of signal abnormality from prior brain MRI but with mildly decreased intensity (Fig. 3). Repeat PET/CT scans and ultrasound of the testes were once again normal without evidence of recurrent testicular cancer or metastases. However, the patient was initiated on cyclophosphamide and lacosamide. Upon discharge, the patient continued with rituximab, cyclophosphamide, lacosamide, and levetiracetam. Now, 8 months after his initial hospitalization, the patient is closely followed by neurology and endocrinology and remains disoriented with fluctuating levels of alertness.

## Discussion

PLE is a rare neurological syndrome that affects limbic structures, including the hippocampus, hypothalamus, and amygdala. It can present in a variety of ways, including cognitive impairment, vertigo, personality changes, visual disturbances, seizures, and memory loss. PLE is often difficult to diagnose since the symptoms usually precede the diagnosis of malignancy, may mimic other complications, and can have a variable presentation. In particular, the presentation of anti-Ma2 PLE often differs from classical paraneoplastic limbic or brainstem encephalitis and may therefore be underrecognized. In addition to the symptoms of classical PLE, anti-Ma2 PLE presents with a wide gamut of other less common clinical features, such as vertical gaze paresis and various endocrine abnormalities.

In the case of our patient, while he did exhibit some of the neurologic symptoms such as cognitive impairment, confusion, vertigo, and visual disturbances characteristic of PLE, his first symptoms were actually endocrine in nature. We thus describe an exceedingly rare case of PLE presenting initially with panhypopituitarism, with weight gain, fatigue, and hypogonadism that began a few months prior to his neurological symptoms. There are only a few cases documenting patients with PLE, particularly anti-Ma2 PLE, who presented with symptoms of panhypopituitarism [3, 4]. While this presentation is rare, it is not unusual for anti-Ma2 PLE to affect the hypothalamus, which could also impact other structures in close proximity, such as the pituitary gland.

PNS precede the diagnosis of cancer by several months in almost 80% of patients, with most tumors diagnosed within 4–6 months of presentation. However, according to official guidelines using the PNS-Care Score, diagnosis of probable PNS should be strongly considered within 2 years of a cancer diagnosis [5]. Our patient’s symptoms of PLE started 1.75 years after his initial testicular cancer diagnosis, not following the most common pattern of PNS. Even though his cancer was no longer active, a high index of clinical suspicion for PNS for any patients with a cancer diagnosis within the last 2 years allowed our patient to undergo appropriate testing to make the diagnosis of PLE and for him to be treated accordingly. This case also serves as a reminder that, in general, paraneoplastic syndromes can occur even after cancer is cured due to their immunologic nature.

Patients who are suspected of having a PNS often undergo whole-body PET to screen for the location of the occult cancer, but searches for the cancer may be unrevealing if the cancer was in the past or has not yet presented [6]. Laboratory findings for those with PNS include mild pleocytosis, slightly elevated protein level, and an elevated immunoglobulin G (IgG) level upon examination of cerebrospinal fluid. One of the most helpful and cutting-edge diagnostic tools is highly specific PNS antibodies in the serum and cerebrospinal fluid of affected persons. In patients with high suspicion of paraneoplastic encephalopathy, common antibodies to test for include anti-Hu, anti-amphiphysin, anti-collapsin response-mediator protein-5 (CRMP5), anti-Purkinje cell antibody 2 (PCA-2), anti-Ma1, and anti-Ma2. Among these paraneoplastic antibodies, anti-Ma2 is the one most commonly associated with testicular cancer in young male patients, as seen in our patient [1]. Timely detection and diagnosis of PNS can lead to prompt initiation of treatment and a better outcome.

Treatment of PNS includes two main approaches: treatment of the underlying tumor and suppression of the immune response. Ablating the tumor and initiating oncological treatment is often the most effective treatment to prevent progression of neurological syndromes [1]. However, if the patient’s condition is worsening rapidly, a combination of plasma exchange, intravenous immune globulin, and immunosuppressive agents such as corticosteroids or cyclophosphamide is used [7]. Due to a paucity of evidence-based data, there are unfortunately no established protocols for immunosuppressive treatment for most paraneoplastic syndromes, and immunotherapy is not always effective. PNS is typically a rapidly destructive immune process with an overall guarded prognosis. While immunotherapy has not been established in studies to be effective for treating most paraneoplastic syndromes, a small number of case reports describing response to immunotherapy regimens encourages physicians to start treatment quickly to dampen the rapidly destructive process of PNS [1, 7].

## Conclusion

We present a case of anti-Ma2 paraneoplastic limbic encephalitis with initial symptoms of panhypopituitarism in a young patient with history of testicular cancer in remission. Anti-Ma2 PLE is a rare but possible etiology of panhypopituitarism. Because of its variable presentation, PLE can be difficult to diagnose, leading to delays. To expedite time to diagnosis and treatment, a high index of suspicion is warranted for paraneoplastic syndromes in patients with history of malignancy, even after the malignancy is no longer active.

## Data Availability

Not applicable.
